# Pyoderma Gangrenosum following Pacemaker Implantation: A Case Report and Review of Literature

**DOI:** 10.1155/2019/8010895

**Published:** 2019-11-20

**Authors:** Noura Ayoubi, Zaydi Javeed, Raymond Cutro, Brooke T. Baldwin

**Affiliations:** ^1^Morsani College of Medicine, University of South Florida, Tampa, Florida, USA; ^2^Department of Cardiology, James A. Haley Veterans' Hospital, Tampa, Florida, USA; ^3^Department of Dermatology, James A. Haley Veterans' Hospital, Tampa, Florida, USA

## Abstract

Pyoderma gangrenosum (PG) is a rare neutrophilic dermatosis characterized by noninfectious, inflammatory, ulcerating lesions. Pathergy can be seen in these patients, whereby minor trauma or surgery can result in the development of PG ulcerations. Here, we present a case of PG following pacemaker implantation. A 76-year-old male with a history of rheumatoid arthritis presented to the cardiology team with symptomatic bradycardia. Indications for implantation were met, and the procedure was performed in a routine fashion. The patient returned to clinic for follow-up four days later, complaining of pain at the incision site, coupled with erythema and purulent drainage. Consultations with an infectious disease specialist and a dermatologist were requested, and the diagnosis of pyoderma gangrenosum was considered. The patient underwent device removal and received systemic corticosteroids at a dose of 1 mg/kg prednisone with complete lesion healing in 3 weeks. While being maintained on steroids, the patient underwent reimplantation of a new pacemaker on the contralateral side without complication and had a normal postoperative course. We present this case report, along with the review of literature, in order to highlight the multidisciplinary approach to management, which requires dermatologic treatment in order to achieve pacemaker success.

## 1. Introduction

Pyoderma gangrenosum (PG) is a rare neutrophilic dermatosis characterized by noninfectious, inflammatory, ulcerating lesions [1]. Lesions occur most commonly on the lower legs, but any part of the skin can be involved. The most common age of presentation is between 40 and 60 years, with less common cases occurring in children or older adults [1, 2]. While the exact etiology is unknown, the neutrophilic reaction is hypothesized to occur due to the interplay between neutrophilic dysfunction, genetic risk factors, and systemic inflammation. Ulcerative colitis, Crohn's disease, rheumatoid arthritis, and chronic active hepatitis are commonly involved inflammatory disorders [1]. Pathergy can be seen in these patients, whereby minor trauma or surgery can result in the development of PG ulcerations [1].

Here, we present a case of PG following pacemaker implantation in a patient with rheumatoid arthritis. This rare complication has only been reported in the literature in 6 cases [3–8]. We will be presenting our case as well as reviewing the literature in order to characterize cases of PG complicating pacemaker implantation and how dermatologic and cardiac conditions were concomitantly managed.

## 2. Case Report

A 76-year-old male with a history of rheumatoid arthritis presented to the cardiology team with symptomatic bradycardia. Indications for implantation of a dual-chamber pacing system were met, and the procedure was performed in a routine fashion in the left prepectoral area by the cardiac electrophysiologist. Standard postoperative precautions were taken, and the patient was discharged uneventfully the following day. A course of prophylactic antibiotics was prescribed, consisting of doxycycline 100 mg PO twice daily.

**Figure 1:**
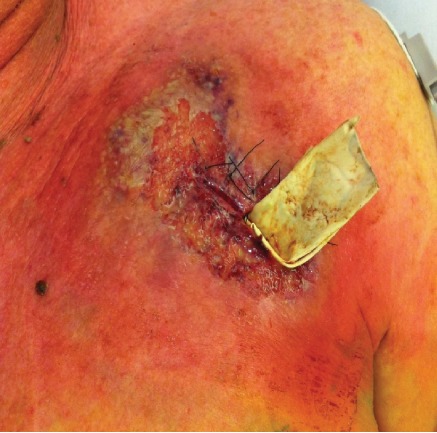
Purulent ulcer with dusky, violaceous borders on a background of erythema.

The patient returned to the pacemaker clinic for follow-up four days later, complaining of pain at the incision site, coupled with erythema and purulent drainage (Figure 1). Signs and symptoms were suspicious for a pocket infection, and the patient was admitted for parenteral antibiotics (vancomycin and piperacillin/tazobactam) and scheduled for device removal. A complete blood count (CBC) revealed a mildly elevated white blood cell count (10.9 × 10^3^/microliter) and a neutrophil count within normal limits. Swabs of the purulent material in the pacemaker pocket at the time of device explantation were sent for aerobic and anaerobic cultures and were negative for bacterial growth. Three sets of blood cultures were also negative.

**Figure 2:**
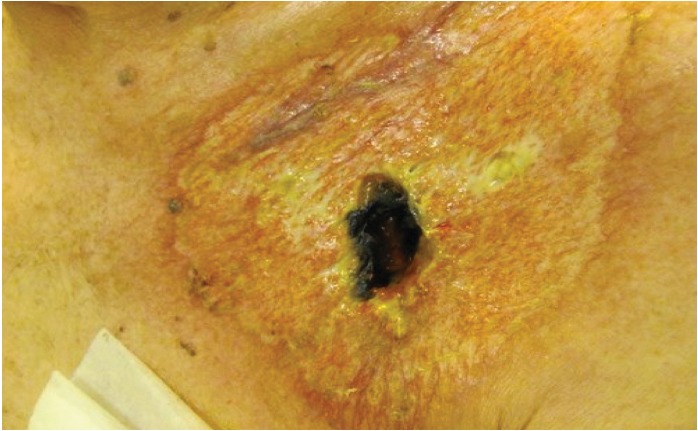
Healing ulcer with black central crusting and a rim of erythema.

Consultations with an infectious disease specialist and a dermatologist were requested, and the diagnosis of pyoderma gangrenosum was strongly considered. Skin biopsy was performed, revealing massive neutrophilic infiltration and necrosis consistent with pyoderma gangrenosum. The patient underwent device removal and received systemic corticosteroids at a dose of 1 mg/kg prednisone with complete lesion healing in 3 weeks (Figure 2). While being maintained on steroids, the patient underwent reimplantation of a new pacemaker on the contralateral side without complication and had a normal postoperative course. Steroids were then tapered gradually over 3 months with no recurrence of disease 1 year later.

## 3. Discussion

PG has been associated with systemic inflammation, surgery, or minor trauma. However, most cases are of unknown etiology. PG lesions often begin as tender, inflammatory papules or pustules with an indurated, dusky red peripheral rim [1]. As necrosis ensues, an ulcer forms a pustular base with a violaceous border. Less commonly, PG can present with bullous, vegetative, or pustular predominance [9]. Postoperative PG can be seen following surgery, whereby ulcers present at the surgical site within 2 weeks following the operation [10]. This presents as severe pain out of proportion to physical exam along with erythema and wound dehiscence [10].

Diagnosis of PG is often difficult to make, given the nonspecific clinical and histopathological manifestations. Clinical findings may mimic primary cutaneous infection, vasculitis, drug-induced tissue injury, or other inflammatory disorders [1]. Biopsy often reveals nonspecific findings that can include granulomatous dermatitis, folliculitis, and leukocytoclastic vasculitis [1]. Workup for underlying etiologies of PG should be considered. This includes CBC, erythrocyte sedimentation rate (ESR), liver function tests (LFTs), protein electrophoresis, antineutrophil cytoplasmic antibodies (ANCA), and cryoglobulins [1]. In many cases, an underlying etiology is not found.

The first-line treatment of PG is systemic corticosteroids (0.5-2.0 mg/kg/day), along with local wound management [1, 8]. Addition of oral cyclosporine to systemic steroids may be required when initial disease is severe [1]. Mycophenolate mofetil, cyclophosphamide, chlorambucil, and azathioprine have also been effective in corticosteroid-resistant cases [1, 11]. Once systemic corticosteroid is tapered, maintenance with immunosuppressant agents can be considered. Commonly used immunosuppressants are cyclosporine and antitumor necrosis factor (TNF) agents, which include infliximab and etanercept [8].

Presentation of PG following pacemaker implantation has been reported in 6 cases, 4 of which were initially managed as infectious complications and treated with antibiotics (Table 1). Following continued necrosis and ulceration, further workup revealed a diagnosis of pyoderma gangrenosum. In all cases, systemic corticosteroids resulted in local improvement of PG ulcers. In some of the cases, reimplantation of the pacemaker with the use of cyclosporine allowed cardiologists to manage the cardiac condition while preventing recurrence of PG.

We present this case report, along with the review of literature, in order to highlight the multidisciplinary approach to management, which requires dermatologic treatment in order to achieve pacemaker success. Given the rare nature of the complication, cardiologists are unlikely to encounter it in practice. However, when it does occur, it is likely that an infectious nature be the highest on the list of differential diagnoses simply due to the clinical presentation. Nevertheless, it is important for cardiologists to consider PG and have a lower threshold for a dermatologic consult or biopsy in cases where antibiotics have been ineffective. This can prevent delayed diagnosis, reducing the risk of more severe complications such as actual infection. Furthermore, prophylaxis against PG can allow for reimplantation of the pacemaker by cardiologists, allowing patients to receive the requirement operation that prompted this condition in the first place.

## Figures and Tables

**Table 1:** Pyoderma gangrenosum following pacemaker implantation.

Author, year	Patient characteristics	Onset	Presentation	Outcome
Lo et al. [3], 2002	85 F with monoclonal gammopathy and polymyalgia rheumatica	Not discussed	Ulcer with erythematous border on the left anterior chest	Initial treatment as infection led to pacemaker extraction; healing occurred with systemic corticosteroids.

Gebska et al. [4], 2005	71 M	Recurrent skin lesions at the site of pacemaker implantation in the upper thorax	Not discussed	Steroid therapy resulted in improvement and abdominal implant was considered instead.

Kaur et al. [5], 2006	71 F with syncope	3 weeks following pacemaker implantation	Red, indurated, and partially open ulcer at the site of implant	Patient was started on antibiotics and pacemaker was removed. Two more trials of pacemaker insertion were done with continued ulceration in both cases. Topical steroids were ineffective. Prednisolone (20 mg/day) for 10 days resulted in improvement. Remained well-controlled on cyclosporine for two months without recurrence.

Cosio et al. [6], 2006	70 F with monoclonal gammopathy, diabetes, and 2 : 1 atrioventricular block	Postoperative day 7	Painful, necrotic lesion at the implant site	Prednisone (60 mg daily) led to healing within 3 weeks. Patient developed concomitant heart failure and nephrotic syndrome, which led to death 1 month following admission.

Duncan et al. [7], 2009	64 M with second-degree heart block	15 months following pacemaker implantation	Unrelated trauma to the implant site led to cystic swelling and subsequent ulceration	Pacemaker broke down following ulceration. Antibiotics failed to benefit. Prednisolone (60 mg/day) led to improvement. This was followed by recurrence after steroids taper. Restarting prednisolone with cyclosporine (3 mg/kg) led to complete healing. Patient remained controlled on cyclosporine.

Marzak et al. [8], 2019	72 M with 2 : 1 atrioventricular block	Postoperative day 4	Inflammatory, infiltrative, necrotic ulcer at the implant site	Initial treatment with antibiotics led to continued necrosis; local healing occurred with systemic corticosteroids. On postoperative day 45, patient developed septic shock secondary to infection of prior PG lesion. Removal and reimplantation of new pacemaker were done. IV infliximab (5 mg/kg), colchicine (1 mg/day), corticosteroids (1 mg/kg), and antibiotics were prophylactically given. One-year follow-up was uneventful.

Ayoubi et al., 2019	76 M with rheumatoid arthritis	Postoperative day 4	Pain at the incision site, coupled with erythema and purulent drainage	Initial treatment with antibiotics was ineffective. Swabs of purulent material revealed negative cultures for bacterial growth. Systemic prednisone (1 mg/kg) resulted in complete healing in 3 weeks. Steroid maintenance allowed for reimplantation of new pacemaker without complications.

## Abbreviations

PG: Pyoderma gangrenosum
CBC: Complete blood count
ESR: Erythrocyte sedimentation rate
LFT: Liver function tests
ANCA: Antineutrophil cytoplasmic antibodies
TNF: Tumor necrosis factor.
